# Depletion of abundant plasma proteins for extracellular vesicle proteome characterization: benefits and pitfalls

**DOI:** 10.1007/s00216-023-04684-w

**Published:** 2023-04-18

**Authors:** Sandrine Reymond, Lyssia Gruaz, Jean-Charles Sanchez

**Affiliations:** Department of Medicine, Faculty of Medicine, University of Geneva, Geneva, Switzerland

**Keywords:** Extracellular vesicles, Plasma depletion, Proteomics, Size exclusion chromatography

## Abstract

Blood extracellular vesicles (EVs) play essential roles in cell–cell communication and their molecular cargo is a promising source of disease biomarkers. However, proteomic characterization of plasma-derived EVs is challenged by the presence of highly abundant plasma proteins, which limits the detection of less abundant proteins, and by the low number of EVs in biological fluids. The aim of this study was to investigate if the removal of abundant plasma proteins prior to EV isolation could improve plasma-derived EV characterization by LC–MS/MS and expand the proteome coverage. Plasma depletion was performed using a single-use spin column and EVs were isolated from only 100 µL of non-depleted and depleted plasma by size exclusion chromatography. Afterwards, EVs were characterized by nanoparticle tracking analysis and mass spectrometry–based proteomics using a data-independent acquisition approach. Depleted plasma-derived EVs had higher particle concentrations and particle-to-protein ratios. Depletion did increase the protein coverage with a higher number of identifications in EVs from depleted plasma (474 proteins) than from non-depleted (386 proteins). However, EVs derived from non-depleted plasma carried a slightly higher number of common EV markers. Overall, our findings suggest that plasma depletion prior to EV isolation by size exclusion chromatography provides higher yield and protein coverage, but slightly lower identification of EV markers. This study also showed the possibility to characterize the proteome of EVs derived from small plasma volumes, encouraging the clinical feasibility of the discovery of EV biomarkers.

**Supplementary Information:**

The online version contains supplementary material available at 10.1007/s00216-023-04684-w.

## Introduction

Extracellular vesicles (EVs) are membrane-enclosed structures, which are secreted by prokaryote and, presumably, all eukaryote cells [1]. They have been shown to be involved in cell–cell communication and in many physiological and pathological processes [2, 3]. They form a heterogenous group associated to the diversity of possible cargoes, physical characteristics, and biogenesis [4]. EVs’ cargo is composed of proteins, lipids, nucleic acids, and metabolites [5]. Their composition is modified depending on pathophysiological conditions in order to deliver specific molecular messages to recipient cells [6].

In the last two decades, the number of publications on EVs has drastically increased and this is also true for studies using mass spectrometry (MS)–based protein analysis [7, 8]. Indeed, high-throughput proteomics is used to investigate the complex protein content of EVs originated from different body fluids and cell types. Moreover, this protein cargo represents a promising source of biomarkers for disease diagnosis and monitoring [5]. Plasma is currently the most studied fluid for EV disease biomarkers, as it allows their detection in the form of a liquid biopsy and can be used in studies utilizing large cohorts and biobanks [5]. However, the low number of EVs makes difficult their investigation in plasma [9]. As a result, EV isolation prior to molecular analysis is key, especially for biomarker discovery using proteomics [10]*.* However, there is a large variety of methods for EV isolation (ultracentrifugation, polymer-based precipitation, or affinity-based isolation), which all have some advantages and disadvantages regarding yield and purity. Therefore, the choice depends on the planned downstream analysis.

Additionally, the high dynamic range of protein abundance in plasma limits the detection of less abundant proteins in proteomics studies [11]. Indeed, around 55% of the total protein mass in plasma is albumin, while 85% corresponds to the seven most abundant proteins together [12]. High-abundant plasma protein depletion is commonly used to increase proteome coverage in global plasma proteomics studies, by allowing the detection of proteins present in lower abundance [12]. Depletion is commonly performed using immunoaffinity with multiple- or single-use depletion columns removing up to 20 abundant plasma proteins [13]. Therefore, the removal of abundant plasma proteins could reduce their interference with the proteomic characterization of EVs [9].

In this study, we aimed to investigate if the removal of abundant plasma proteins prior to EV isolation could improve plasma-derived EV characterization by LC–MS/MS and expand the proteome coverage. To do so, plasma depletion was performed using a single-use spin column prior to size exclusion chromatography (SEC)–based EV isolation. SEC has been shown to reach a higher yield than ultracentrifugation and to perform better in limiting the co-isolation of soluble plasma proteins in comparison to polymer-based precipitation methods [14–17]. In addition, SEC is suitable for small volumes, allowing us to isolate EVs from 100 µL, which is highly relevant regarding clinical samples stored in biobanks. Afterwards, EVs derived from non-depleted and depleted plasma were characterized by nanoparticle tracking analysis (NTA) and mass spectrometry (MS)–based proteomics using a data-independent acquisition (DIA) approach. Improvement of proteomic analyses of EVs is an essential step to promote biomarker discovery.

## Materials and methods

### Plasma collection and preparation

Blood samples were collected from two healthy and fasting laboratory workers at the University of Geneva in K_2_EDTA tubes and were anonymized. To obtain plasma, blood was centrifugated twice at 2500 × g for 10 min at room temperature within the hour following blood collection. Plasma supernatant was recovered, pooled, aliquoted, and frozen at − 80 °C. Plasma aliquots (100 µL) were thawed on ice. Depletion was performed on plasma aliquots (100 µL) using High Select™ Top14 Abundant Protein Depletion Midi Spin Columns (Thermo Scientific, Waltham, MA, USA) according to the manufacturer’s instructions. Filtrate was recovered and concentrated using Amicon Ultra-2 Centrifugal Filter 100 kDa MWCO (Millipore).

### EV isolation using size exclusion chromatography

Depletion was performed on three plasma aliquots (100 µL) and the filtrate was recovered and concentrated until a volume of 100 µL was reached. Afterwards, the three depleted plasma samples and three additional non-depleted plasma (100 µL) were centrifuged at 10,000 × g for 10 min. EVs from depleted and non-depleted plasma were isolated by size exclusion chromatography (SEC) on qEV single 70 nm columns (iZON Science, Christchurch, New Zealand) according to the manufacturer’s instructions. Briefly, SEC columns were equilibrated at room temperature and flushed with 6 mL of phosphate buffer saline (PBS) before use. Plasma was loaded onto the column. Fifteen to sixteen 200 µL fractions were collected and kept at 4 °C overnight.

### Nanoparticle tracking analysis

Particle concentration and size distribution were determined by nanoparticle tracking analysis (NTA), using a Particle Metrix ZetaView® instrument (Particle Metrix GmbH, Inning, Germany). EVs were diluted with 0.2 μm-filtered PBS prior to analysis to reach optimal particle concentration (20–200-fold dilution). Data acquisition in scatter mode was performed using a laser wavelength of 520 nm and standard instrument settings (sensitivity: 80; shutter: 100; minimum brightness: 30; minimum area: 10; maximum area: 1000). Data acquisition in fluorescent mode was performed using a laser wavelength of 660 nm and standard instrument settings (sensitivity: 94; shutter: 100; minimum brightness: 20; minimum area: 10; maximum area: 1000). For fluorescent mode, EVs were stained with CellMask™ Deep Red plasma membrane stain (Invitrogen, Waltham, MA, USA). Data were analyzed with ZetaView® software version 8.05.12 SP1 (Particle Metrix GmbH, Inning, Germany). Afterwards, EVs were dry-stored at − 80 °C.

### Protein preparation and quantification

EV pellets were resuspended in 80 µL of 0.1% Rapigest SF Surfactant (Waters, Milford, MA, USA) 100 mM TEAB (Sigma-Aldrich, St. Louis, MO, USA). EV samples and crude plasma (1:100 dilution) were incubated for 10 min at 80 °C and sonicated for 5 cycles of 20 s with breaks on ice. After centrifugation at 14,000 × g for 10 min at 4 °C, the supernatant was recovered. Protein content was measured using the Bradford assay (Bio-Rad, Hercules, CA, USA).

### Sample preparation for LC–MS/MS

Each sample (plasma samples (1 µg) and EV samples (70 µL)) was reduced using TCEP 0.1 M (final concentration 5 mM, 30 min, 37 °C) (Sigma-Aldrich), alkylated using iodoacetamide 150 mM (final concentration 15 mM, 60 min, RT, in dark condition) (Sigma-Aldrich), and digested by an overnight tryptic digestion (w/w ratio 1:50, 37 °C) (Promega). The RapiGest surfactant was cleaved by incubating samples with 0.5% trifluoacetic acid (45 min, 37 °C) (Sigma-Aldrich). Samples were desalted on a C18 reverse phase column (Harvard Apparatus) and the remaining peptides were dried in Savant SPD111V SpeedVac Concentrator (Thermo Fisher). They were stored at − 80 °C and, prior to MS injection, they were resuspended in 5% ACN 0.1% FA with the addition of iRT peptides (ratio 1:20) (Biognosys).

### Data-independent acquisition mass spectrometry (DIA MS) and data analysis

Plasma samples to assess the efficacy of plasma depletion were diluted in 10 μL of loading buffer (5% CH_3_CN, 0.1% FA) and 4 μL were injected on-column. LC–ESI–MS/MS was performed on a Q-Exactive HF Hybrid Quadrupole-Orbitrap Mass Spectrometer (Thermo Fisher Scientific) equipped with an Easy nLC 1000 liquid chromatography system (Thermo Fisher Scientific). Peptides were trapped on an Acclaim pepmap100, C18, 3 μm, 75 μm × 20 mm nano trap-column (Thermo Fisher Scientific) and separated on a 75 μm × 250 mm, C18, 2 μm, 100 Å Easy-Spray column (Thermo Fisher Scientific). The analytical separation was run for 125 min using a gradient of H2O/FA 99.9%/0.1% (solvent A) and CH3CN/FA 99.9%/0.1% (solvent B). The gradient was run from 8% B to 28% B in 105 min, then to 42% B in 20 min, then to 95% B in 5 min with a final stay of 20 min at 95% B. The flow rate was 250 nL/min and the total run time was 150 min. Data-independent acquisition (DIA) was performed with MS1 full scan at a resolution of 60,000 (FWHM) followed by 30 DIA MS2 scan at a resolution of 30 (FWHM) with 28 m*/z* isolation width within an *m/z* range of 400 to 12,400. MS1 was performed with an AGC target of 3 × 10^6^ and a maximum injection time of 60 ms. DIA MS2 was performed using higher-energy collisional dissociation (HCD) at 27%, an AGC target of 1 × 10^6^, and a maximum injection time of 50 ms.

EV and plasma samples were diluted in 10 μL of loading buffer (5% CH_3_CN, 0.1% FA) and 4 μL were injected on-column. LC–ESI–MS/MS was performed on Orbitrap Fusion Lumos Tribrid Mass Spectrometer (Thermo Fisher Scientific). Settings were identical to those used for MS acquisitions in [18]. Data-independent acquisition (DIA) was performed with MS1 full scan at a resolution of 60,000 (FWHM) followed by 30 DIA MS2 scans with 28 m*/z* isolation width within an *m/z* range of 400 to 1240. MS1 was performed with an AGC target of 1 × 10^6^ and a maximum injection time of 50 ms. DIA MS2 was performed using higher-energy collisional dissociation (HCD) at 27%. Isolation windows were set to 28 m*/z* with an AGC target of 1 × 10^6^ and a maximum injection time of 54 ms.

DirectDIA analysis workflow was used in Spectronaut™ (Biognosys AG, Zurich, Switzerland) to match DIA MS raw data. Carbamidomethyl was defined as a fixed modification and oxidation of methionine as a variable modification. Protein and PSM false discovery rate were set to 0.01 and data filtering was set to *Q*-value. For the analysis of plasma and EVs, protein identifications were exported from Spectronaut™. The mass spectrometry proteomics data have been deposited to the ProteomeXchange Consortium via the PRIDE [19] partner repository with the dataset identifier PXD039240.

### EV protein database searches

The list of top 100 proteins identified in EVs according to Vesiclepedia was obtained through FunRich 3.1.3 and according to ExoCarta on the website (http://exocarta.org/).

### Gene ontology analysis

Gene ontology (GO) enrichment analysis for cellular components and biological processes was performed using Metacore™ (Clarivate Analytics, Philadelphia, PA, USA).

### Statistics

Statistical analysis was performed with Student’s unpaired *t*-test. A *p*-value < 0.05 was considered statistically significant. Venny 2.1 was used to compare datasets (BioinfoGP, CNB-CSIC). Hierarchical clustering analysis and heatmap were generated in R (version 4.2.1) with functions kmeans and pheatmap respectively. Graphical representations were prepared using GraphPad Prism version 9.4.1 for Windows (GraphPad Software, San Diego, CA USA), GIMP version 2.10.30 (https://www.gimp.org/), and BioRender (https://biorender.com/).

## Results

### Depletion of abundant plasma proteins

In this study, we aimed to investigate if the removal of abundant plasma proteins prior to EV isolation using SEC could improve the molecular coverage of plasma-derived EV proteome. Fasting plasma was obtained from two healthy donors. In order to deplete rapidly and in a single step 100 µL of plasma, we selected the High Select™ Top14 Abundant Protein Depletion Midi Spin Columns (Thermo Scientific). Then, the recovered filtrate was concentrated by Amicon Ultra-2 Centrifugal Filter 100 kDa MWCO (Millipore).

To assess the efficiency of the depletion, mass spectrometry analysis was performed on plasma before and after depletion. We examined the top 20 most abundant proteins in both depleted and non-depleted plasma samples (see Electronic Supplementary Material (EMS) 1, Fig. S1). In comparison to the top 20 of non-depleted plasma, alpha-1-antitrypsin (SERPINA1), alpha-2-macroglobulin (A2M), fibrinogen (FGA, FGB, FGG), haptoglobin (HP), serotransferrin (TF), and several immunoglobulins (IGHA1, IGHG1, IGHG2, IGKC and IGHM) were removed from the top 20 of depleted plasma, while apolipoprotein A-I (APOA1) and albumin (ALB) were partially removed, demonstrating that the depletion for these abundant plasma proteins was efficient.

### Characterization of SEC-isolated EVs derived from non-depleted and depleted plasma

**Fig. 1:**
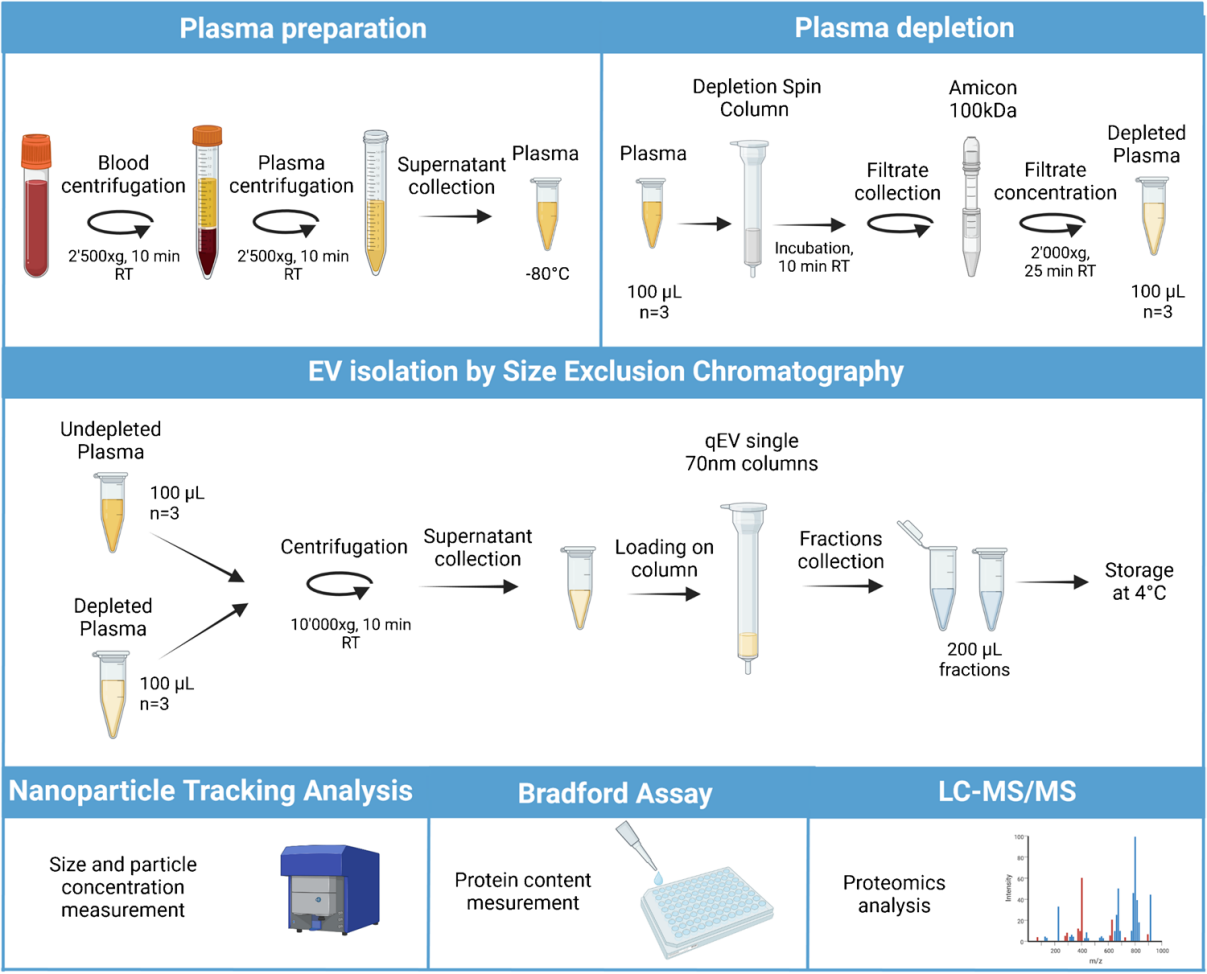
Schematic diagram of the protocol used to isolate and characterize plasma-derived EVs. Figure created using BioRender

Before EV isolation, depletion was performed on three plasma aliquots (100 µL) and the filtrate was recovered and concentrated up to 100 µL. Afterwards, EV isolation was performed on the three depleted plasma samples and three additional non-depleted plasma (100 µL) using SEC, and fractions of 200 µL were collected (Fig. 1).

To evaluate the EV elution profile, particle concentration and size were measured by nanoparticle tracking analysis (NTA) in scatter mode (s-NTA) for each individual SEC fraction. For both EV samples, the elution profile showed peaks in particle concentrations in fractions 6–8, suggesting that these fractions contained the EVs (EMS1, Fig. S2).

Afterwards, protein and particle concentration of pooled SEC fractions (F1–F5, F6–F8, and two-by-two for F9 to F15) were analyzed to determine which fractions had the highest number of particles and the least amount of protein (EMS1, Fig. S3). Particle concentrations peaked in the pooled fraction F6–F8, corresponding to the elution of EVs. On the contrary, protein amount rose in later fractions (from F13 onwards), demonstrating that most plasma proteins eluted later. Interestingly, the protein amount was lower in later fractions from depleted plasma than in non-depleted plasma, supporting the removal of abundant plasma proteins during depletion. Moreover, the particle-to-protein ratio was the highest for the presumed EV-containing fractions 6–8 corresponding to a high particle and low protein content (data not shown). These results indicated that SEC was able to effectively separate EVs from a high proportion of soluble plasma proteins. For all further analyses, these fractions were collected, pooled, and referred to as N EVs for EVs derived from non-depleted plasma and D EVs for EVs derived from depleted plasma. In addition, the particle-to-protein ratio of D EVs was statistically higher than the ratio of N EVs (6.67 × 10^10^ ± 2.89 × 10^9^ and 4.4 × 10^9^ ± 2.02 × 10^8^, respectively, *p*-value < 0.0001), suggesting a higher enrichment with a reduced non-EV protein presence [10].

**Fig. 2:**
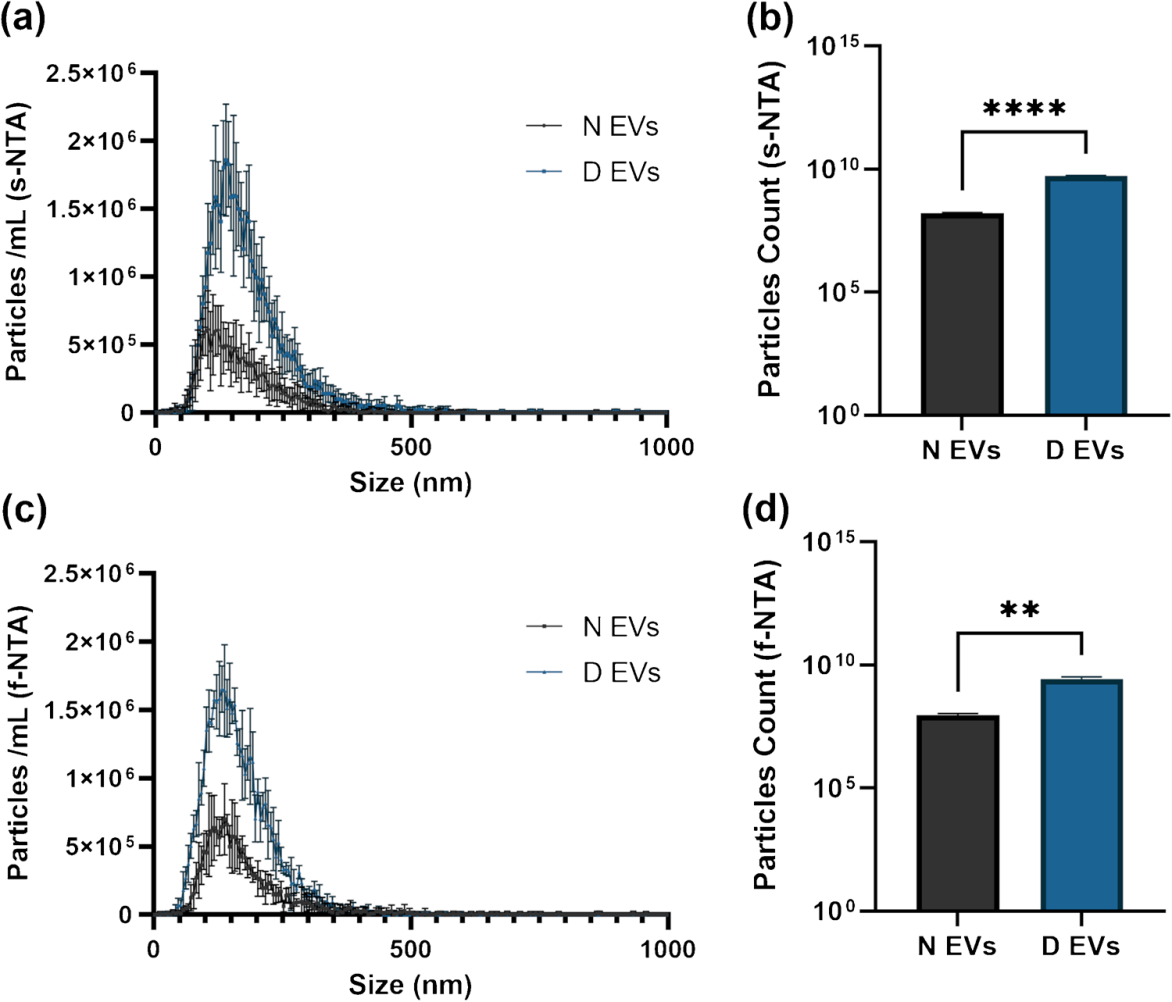
Size distribution (**a**) and particles count (**b**) characterization of extracellular vesicles isolated from non-depleted plasma (N EVs) and depleted plasma (D EVs) by nanoparticle tracking analysis in scatter mode (s-NTA). Size distribution (**c**) and particles count (**d**) of extracellular vesicles isolated from non-depleted plasma (N EVs) and depleted plasma (D EVs), after staining with CellMask Deep Red, by fluorescent nanoparticle tracking analysis (f-NTA). Bars represent mean ± SD. **(a)** *n* = 9; **(b**, **c**, **d)** *n* = 3. Differences with **p-value < 0.05 and ****p-value < 0.0001 were considered statistically significant by *t*-test. Figure created with GIMP

To analyze particle concentration and size of N and D EVs, a combination of NTA in scatter and fluorescent modes was used. As s-NTA measures the total particle count, without differentiating EVs from other light-scattering components, such as lipoproteins and protein aggregates, measurements were also performed in fluorescent mode (f-NTA) after staining of N and D EVs with a membrane dye [20]. S-NTA displayed a slightly increased particle size for D EVs in comparison to N EVs (D EVs: 160.53 ± 1.16; N EVs: 146.28 ± 4.20; *p* = 0.0114), which was not confirmed by f-NTA particle size (D EVs: 150.33 ± 1.76; N EVs: 150.2 ± 2.14; *p* = 0.9376). On the other hand, s-NTA revealed higher particle counts in D EVs (D EVs: 5.27 × 10^9^ ± 4.60 × 10^8^; N EVs: 1.62 × 10^8^ ± 3.85 × 10^8^; *p* < 0.001) (Fig. 2a and b), which was validated by f-NTA measurements (D EVs: 2.62 × 10^9^ ± 1.51 × 10^8^; N EVs: 1.02 × 10^8^ ± 3.12 × 10^7^; *p* = 0.015), suggesting an enhanced recovery of EVs with SEC isolation from depleted plasma (Fig. 2c and d). Of all particles measured in scatter mode, 29.31% and 29.77% were measured in the fluorescent mode for N EVs and D EVs, respectively.

### Proteomics characterization of EV enrichment from non- and depleted plasma

**Fig. 3:**
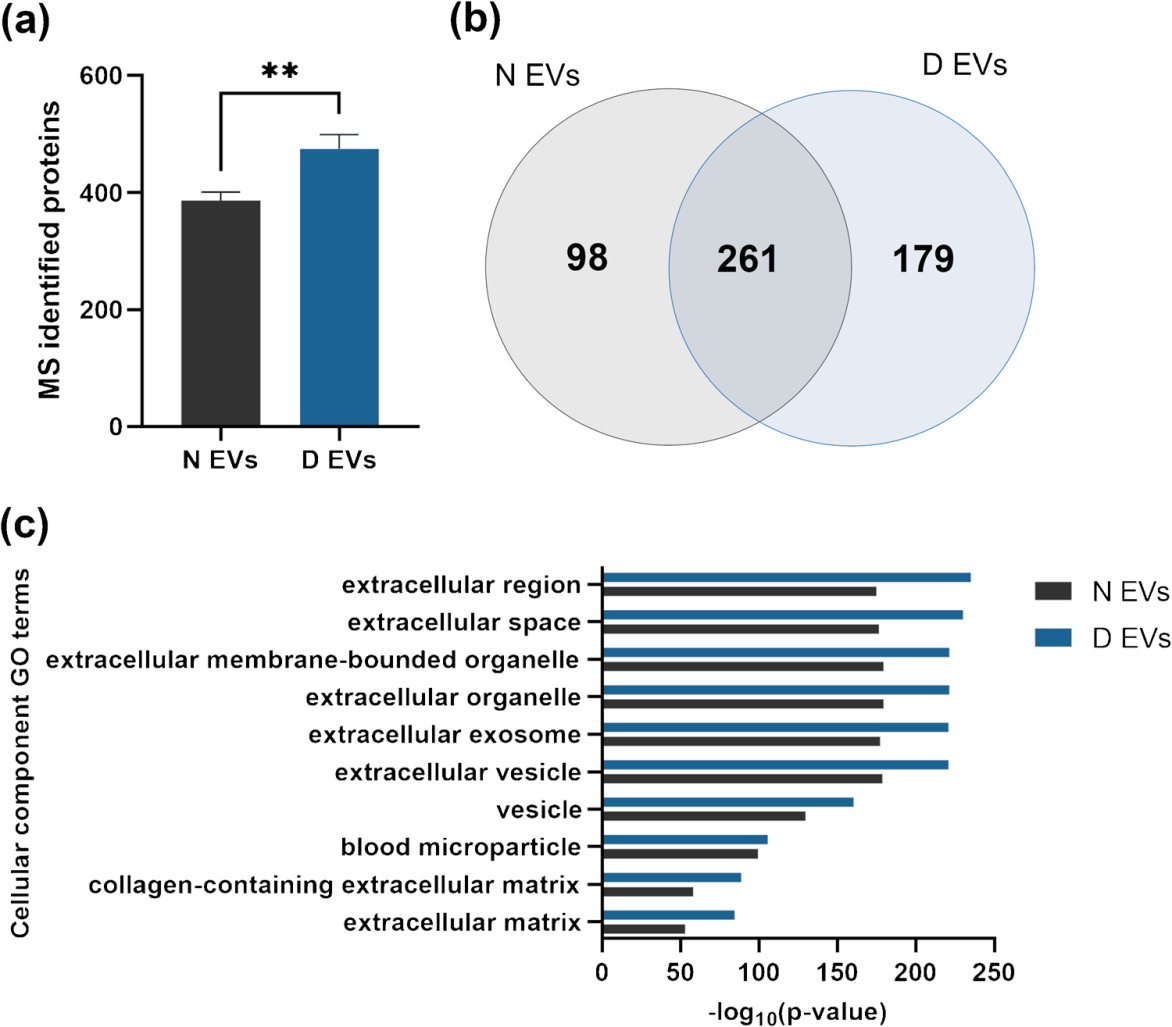
**(a)** Number of identified proteins by mass spectrometry for extracellular vesicles isolated from non-depleted plasma (N EVs) and depleted plasma (D EVs). Bars represent mean ± SD; *n* = 3. Unpaired *t*-test, **p-value = 0.0076. **(b)** Venn diagram of identified proteins for N and D EVs in all replicates (*n* = 3). **(c)** Top 10 enriched GO terms for cellular components after GO analysis with MetaCore software on quantified proteins in N and D EVs. Cellular component GO terms are represented in *y*-axis, while *x*-axis corresponds to -log_10_(*p*-value). The *p*-value cut-off was set at 0.05. Figure created with GIMP

To investigate the impact of plasma depletion on the EV proteome, we performed a DIA-based proteomics analysis for EVs derived from non-depleted and depleted plasma. It resulted in the identification of 386 proteins for N EVs (359 present in all replicates) and 474 proteins for D EVs (440 present in all replicates) (EMS2, Table S1). The number of identified proteins was significantly higher in D EVs than in N EVs, demonstrating an increased protein coverage (Fig. 3a). In addition, both EV samples statistically contained an increased number of identified proteins in comparison to plasma samples, in which 224 proteins were identified (EMS1, Fig. S4). Interestingly, 261 proteins were identified in both EV samples, while 98 proteins were uniquely identified in N EVs and 179 only in D EVs (Fig. 3b).

To observe how depletion impacted the highly abundant plasma proteins in EVs, we looked at the top 20 quantified proteins in N and D EVs (EMS1, Fig. S5). Among the proteins that should be depleted by the top 14 columns, several presented a significative lower abundance in D EVs than in N EVs, as shown above.

Then, we further investigated the proteomic profiling of both EV samples. To do so, only proteins identified in all replicates of each EV sample were conserved for analysis. First, a gene ontology (GO) cellular component enrichment analysis was conducted with the software MetaCore. The top 10 enriched terms included extracellular region, extracellular space, and extracellular membrane-bounded organelle (Fig. 3c), indicating an enrichment of EVs from non-depleted and depleted plasma.

**Fig. 4:**
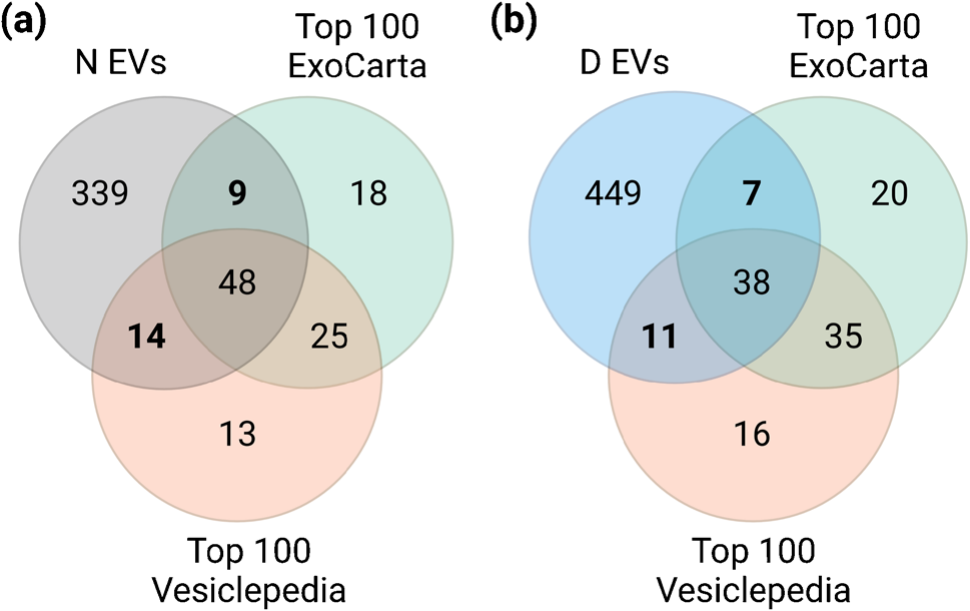
Venn diagrams of identified proteins for **(a)** N EVs and **(b)** D EVs and proteins from top 100 of most often identified proteins in ExoCarta and Vesiclepedia. Figure created with BioRender

Then, the EVs enrichment was evaluated through their protein composition. First, we compared the proteins identified in N and D EVs to EV-associated proteins and non-EV co-isolated components, reported in the Minimal Information for Studies of EVs (MISEV2018) [21] (EMS1, Fig. S6). Interestingly, N EVs carried a slightly higher number of EV-associated proteins in comparison to D EVs (28 and 24 proteins respectively, *p*-value = 0.013). Regarding the co-isolation of non-EV structures, there was no difference between both EV samples in the number of proteins (14 for both), suggesting that plasma depletion prior to EV isolation does not affect the co-isolation of common non-EV components. In addition, we compared the identified proteins in N and D EVs to the top 100 of the most often identified proteins in ExoCarta and Vesiclepedia [22, 23] (Fig. 4). N EVs contained a higher number of “top 100” EV proteins from both databases than D EVs (*p*-value = 0.0042 and *p*-value = 0.0085, respectively). Nonetheless, more than 70% of these proteins were shared by N and D EVs, demonstrating a high overlap between both EV samples.

Our results were also compared to two other studies, which identified more than 1000 proteins from plasma EVs and were relevant regarding their methodology. The study of Karimi et al. [24] investigated the combination of ultracentrifugation, iodixanol density cushion, and SEC to isolate plasma-derived EVs while limiting the co-isolation of lipoproteins and plasma proteins. Even if the initial volume of plasma was very large (40–80 mL), it provided a dataset of highly purified plasma-derived EVs. Secondly, Vanderboom et al. [25] evaluated the performance of SEC coupled to high-resolution MS for comprehensive proteomic analysis of plasma-derived EVs. Among the several experiments of this study, we selected the dataset of EVs isolated from 2 mL of platelet-poor plasma, as it fitted best our experimental design.

**Fig. 5:**
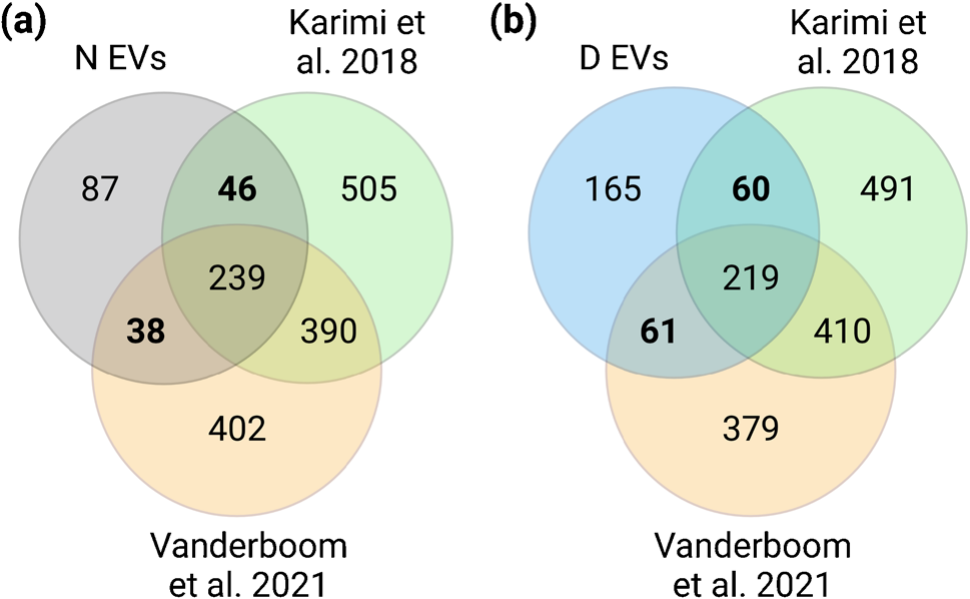
Venn diagrams of identified proteins for **(a)** N EVs and **(b)** D EVs and plasma-derived EV proteomes from the study of Karimi et al. [24] and Vanderboom et al. [25]. Figure created with BioRender

Comparing our data to these two studies, we observed that the majority of proteins in N and D EVs had been identified in at least one of the datasets (N EVs: 75.8%; D EVs: 62.5%) (Fig. 5). This high similarity to these studies, which were performed with different and meticulous EV isolation methods, confirmed the quality of our EV enrichment and proteomic results. Nonetheless, a proportion of proteins in N and D EVs were not identified in the previous studies. These subsets of proteins contained only 3 proteins from the top 100 EV proteins and were mainly blood proteins, keratins, and adhesion proteins. In fact, even if the datasets of Karimi et al. [24] and Vanderboom et al. [25] shared more than half of the identified proteins (53.3% and 58.8%, respectively), an important proportion of proteins was unique to each study (42.7% and 37.6%, respectively) suggesting heterogeneity of EV proteome due to the study design.

**Fig. 6:**
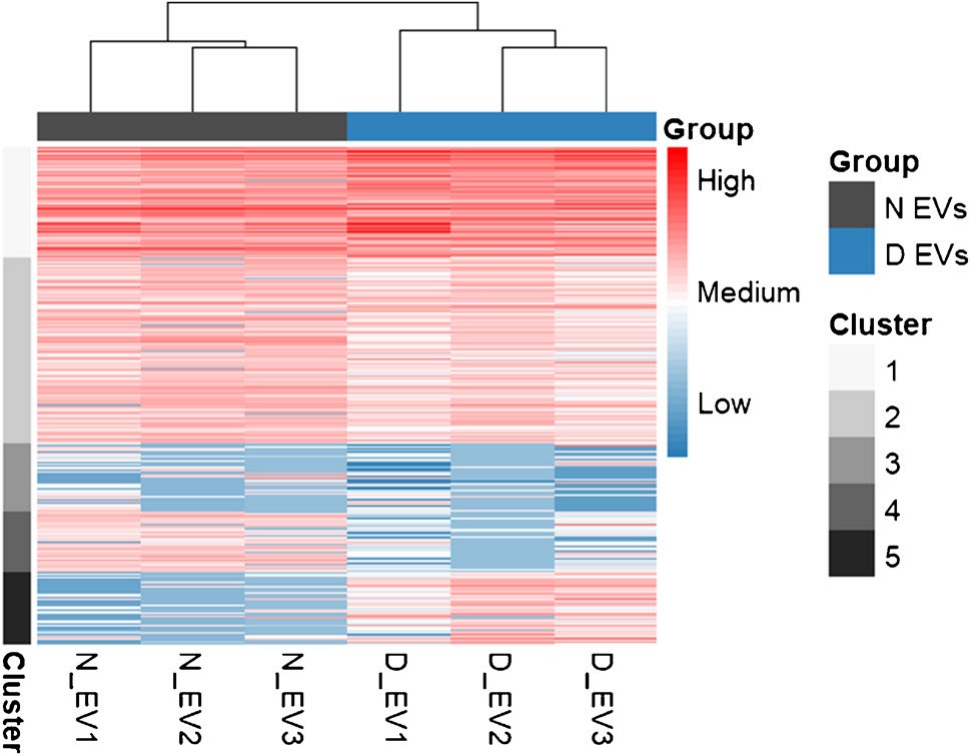
Hierarchical clustering analysis of plasma-derived EVs based on protein abundance and performed with the function kmeans in R. Resulting kmeans values were then represented in an intensity heatmap using the function pheatmap. Rows correspond to proteins and columns to EV sample replicates. The colors represent kmeans values

To further characterize and compare the cargo of EVs obtained from non-depleted and depleted plasma, we performed a hierarchical clustering analysis. Each cluster was mapped to biological process GO terms using MetaCore software (EMS2, Table S2). All replicates of each EV type clustered together, demonstrating good inter-replicate reproducibility (Fig. 6). The majority of proteins exhibited similar abundance between N and D EVs (clusters 1 to 3). Cluster 1, which mainly contained highly abundant plasma proteins, was associated to terms such as “humoral immune response” and “defense response” and cluster 2 to terms like “cell adhesion,” “response to stress” (e.g., due to an injury or infection), and “coagulation.” Such GO terms are common in blood-derived EVs, as they have been described to be involved in coagulation regulation [26, 27] and immune response [3]. Cluster 3 regrouped low-abundance proteins, which were enriched for “keratinization,” resulting from the presence of keratin proteins from an exogenous source, and “intermediate filament cytoskeleton organization,” which mediates vesicle secretion [28].

Interestingly, two subsets of proteins presented differential abundance for N and D EVs. Cluster 4 was composed of proteins enriched in N EVs and which were associated to GO process terms related to the actin cytoskeleton. On the other hand, proteins enriched in D EVs and forming cluster 5 revealed terms linked to the immune response, such as “proteolysis” (which relates to microbial response), “humoral immune response,” and “complement activation.” These results suggested that the proteome of EVs derived from depleted and non-depleted plasma was in the high majority similar with some differences in subsets of proteins (12% and 15% for clusters 4 and 5 respectively).

## Discussion

Plasma-derived EVs are considered a promising source of biomarkers. Despite being a popular biofluid for EV research, plasma and its high dynamic range of protein abundance is a challenging matrix for proteomics studies and the discovery of novel biomarkers.

Therefore, we investigated whether plasma depletion of abundant proteins prior to EV isolation could increase the protein coverage by LC–MS/MS analysis, allowing an improved characterization of EVs. To do so, we compared SEC-isolated EVs derived from only 100 µL of non-depleted and depleted plasma. As a matter of fact, a volume as small as 100µL represents a technical challenge for EV research in terms of volume, but is highly relevant regarding clinical feasibility and biobanks.

To be able to deplete 100 µL of plasma in a single step, we chose the High Select™ Top14 Abundant Protein Depletion Midi Spin Columns (Thermo Scientific). We were encouraged in this choice by the study of Cao et al. [12] which demonstrated that the proteome coverage of these single-use spin columns is comparable to traditional multiple-use HPLC columns and that they provide a simple, reproducible, and cost-effective alternative. Nonetheless, we verified and confirmed by MS analysis that the removal of targeted abundant plasma proteins was successful, as they were among the 20 most abundant proteins in crude plasma, but were mainly absent from the top 20 of depleted plasma. This was also supported by the lower protein content measured in later SEC fractions from depleted plasma.

Regarding EVs, we also observed a higher particle-to-protein ratio for D EVs than N EVs, suggesting an enhanced particle enrichment and lower soluble protein content. As the same number of common co-isolated non-EV structures was identified in N and D EVs, we could conclude that plasma depletion prior to EV isolation led to the partial removal of abundant plasma proteins and did not add non-EV proteins.

Moreover, regarding our initial hypothesis, we demonstrated that plasma depletion prior to EV isolation did increase the protein coverage with a statistically significant increase of identification in D EVs in comparison to N EVs. To the best of our knowledge, few studies explored the effect of depletion on plasma-derived EVs. Interestingly, the study of Diaz Lozano et al. [29] investigated the impact of the removal of three high-abundant plasma proteins (albumin, serotransferrin, and IgG) on the proteome profiling of plasma-derived EVs in a non-obese diabetic mouse. They concluded that plasma depletion of these three proteins enhanced the protein coverage in crude plasma, but not in plasma-derived EVs. This difference may come from the reduced number of targeted proteins for depletion or that depletion was performed directly on plasma-derived EVs, after their isolation with SEC. Similarly, in the study of Palviainen et al. [5], human plasma-derived EVs were depleted of albumin in view of LC–MS/MS analysis. In their case, albumin depletion led to the identification and quantification of 138 proteins instead of 91, showing a higher protein coverage. Therefore, the performance of plasma depletion might depend on the workflow of the study.

Furthermore, we observed the highest particle concentration in D EVs than in N EVs, which was first measured in s-NTA and confirmed in f-NTA for membrane-dyed particles. This suggested that this increase was not caused by non-EV light-scattering components, such as lipoproteins and protein aggregates, which are not differentiated from EVs in scatter mode. This may indicate that the removal of soluble plasma proteins could modify the interaction that EVs have with the SEC matrix, giving rise to an improved enrichment. On the contrary, the increased size measured in s-NTA for D EVs in comparison to N EVs was not confirmed by f-NTA, suggesting a bias from non-EV light-scattering components. In addition, in our study, the particle concentration measured in s-NTA was largely overestimated with only 30% being also measured in fluorescent mode. These findings highlight the importance of the differential use of NTA in scatter and fluorescent mode within samples like plasma.

To assess the EV enrichment in both samples, we explored several strategies to characterize EVs by their protein content based on MS analysis. Interestingly, the number of classical EV markers from ISEV, as well as of the two top 100 most often identified proteins, was slightly higher in N EVs than in D EVs. This suggests that the NTA-measured increase of particle concentration in D EVs did not result in a higher number of EV markers. This finding is a drawback for the use of plasma depletion and challenges the NTA results. To confirm these results, particle concentrations should be analyzed using alternative methods, such as resistive pulse sensing (RPS) or high-resolution flow cytometry, even if each platform has its own concentration and size range for accurate quantitation [21]. Alternatively, antibody-based assays could assess if EV isolation from non-depleted and depleted plasma resulted in differences in EV populations, which could explain the presence of different common EV markers. However, the lack of specific markers of EV subtypes limits these analyses [21].

Additionally, the hierarchical clustering analysis highlighted subsets of proteins with differential abundance between N and D EVs, which were related to different biological processes. Therefore, even if EVs derived from non-depleted and depleted plasma mainly shared the same proteome, the additional step of depletion resulted in some differences in subsets of proteins. Replication of these findings would be required to verify if this is indeed a reproducible and significant modification of the plasma EV proteome due to depletion. Actually, heterogeneity of plasma EV proteomes among different studies has already been reported [24, 30]. Indeed, de Menezes-Neto et al. [30] compared the proteome of human plasma EVs identified in their research to three other studies and noted that of all combined proteins, 49.6% were detected only once. In our study, the proportion of proteins in N and D EVs which were not shared by the datasets of Vanderboom and colleagues [25] and Karimi et al. [24] was 24.2% and 37.5%, respectively. In addition, an important proportion of proteins was also unique to either Vanderboom et al. [25] or Karimi et al. [24] (42.7% and 37.6%, respectively), supporting the report of high heterogeneity. This variability most likely results from the variety of methods for EV isolation, sample preparation, and mass spectrometry techniques, as well as pre-analytics [31–33].

Regarding the technological progress of LC–MS/MS analysis, in the past decade, proteomics studies of plasma-derived EVs struggled to identify more than 250 proteins from initial plasma volumes ranging from one to several dozen of mL [5, 30, 34]. As mentioned previously, in 2018, Karimi et al. [24] reached the identification of 1187 proteins through a combination of three isolation methods. However, it required an initial volume of plasma equivalent to 40–80 mL, as the MS analysis of EVs isolated from 1 mL resulted in only 88 proteins. These last two years, several papers increased the coverage of plasma EV proteome. Of note, Vanderboom and colleagues [25] developed a SEC-MS approach, which identified almost 1300 proteins in EVs derived from 2 mL of plasma and more than 2700 proteins when participants performed aerobic exercise. In 2022, a novel study by Karimi et al. [35] identified 2395 proteins in EVs derived from 7 mL of plasma using immunoaffinity-based isolation. In our study, we successfully identified almost 500 proteins in EVs derived from only 100µL of plasma. We believe that these findings represent a promise for the clinical feasibility of the research of EV biomarkers. Indeed, biomarker discovery and validation are most of the time performed using samples from biobanks and are usually stored in 300 to 500 µL volumes. This would allow performing quantitative biomarker research on a large number of individual samples.

In summary, our study demonstrated that the removal of highly abundant plasma proteins prior to EV isolation resulted in an increase in protein coverage in LC–MS/MS analysis. Interestingly, plasma depletion led to an increased number of particles derived from depleted plasma according to s- and f-NTA, without resulting in an enhanced identification of common EV markers. If it is confirmed by alternative quantification methods, this complexifies the decision to use plasma depletion for proteomics EV characterization and would require deeper analysis to understand the cause of this divergence in common EV markers. Moreover, our research presented several limitations. Firstly, only one method for plasma depletion and EV isolation was tested. Indeed, it has been reported that the chosen EV isolation method will impact the investigated EV subpopulation and its cargo, thus influencing the omics findings [6]. In addition, as pre-analytical conditions also have an impact on the EV composition and amount, our findings should be replicated in different sample types [5, 9, 31]. Secondly, additional orthogonal techniques, such as flow cytometry, could have been used to characterize more thoroughly the EV samples. Nonetheless, the opportunity to improve protein coverage, especially from low plasma volumes, is a great prospect for the discovery of biomarkers in EVs. In addition to the clinical feasibility thanks to low volume, it could allow the detection of low-abundance biomarkers which are otherwise masked by abundant plasma proteins [29].

## Supplementary Information

Below is the link to the electronic supplementary material.

Supplementary file1 (PDF 440 KB)

Supplementary file2 (XLSX 61.1 KB)

## References

[1] Gassama Y, Favereaux A (2021). Emerging roles of extracellular vesicles in the central nervous system: physiology, pathology, and therapeutic perspectives. Front Cell Neurosci.

[2] Tkach M, Théry C (2016). Communication by extracellular vesicles: where we are and where we need to go. Cell.

[3] Buzas EI. The roles of extracellular vesicles in the immune system. Nat Rev Immunol. 2022;1–15. 10.1038/s41577-022-00763-8 10.1038/s41577-022-00763-8 PMC9361922 35927511

[4] Ciferri MC, Quarto R, Tasso R (2021). Extracellular vesicles as biomarkers and therapeutic tools: from pre-clinical to clinical applications. Biology.

[5] Palviainen M, Saraswat M, Varga Z, Kitka D, Neuvonen M, Puhka M, Joenväärä S, Renkonen R, Nieuwland R, Takatalo M, Siljander PRM. Extracellular vesicles from human plasma and serum are carriers of extravesicular cargo—implications for biomarker discovery. PLOS ONE. 2020 15:e0236439. 10.1371/journal.pone.0236439 10.1371/journal.pone.0236439 PMC7446890 32813744

[6] Rosa-Fernandes L, Rocha VB, Carregari VC, Urbani A, Palmisano G. A perspective on extracellular vesicles proteomics. Front Chem. 2017;5. 10.3389/fchem.2017.00102 PMC5702361 29209607

[7] Pocsfalvi G, Stanly C, Vilasi A, Fiume I, Capasso G, Turiák L, Buzas EI, Vékey K (2016). Mass spectrometry of extracellular vesicles. Mass Spectrom Rev.

[8] Couch Y, Buzàs EI, Di Vizio D, Gho YS, Harrison P, Hill AF, Lötvall J, Raposo G, Stahl PD, Théry C, Witwer KW, Carter DRF. A brief history of nearly EV‐erything – the rise and rise of extracellular vesicles. J of Extracellular Vesicle. 2021;10. 10.1002/jev2.12144 10.1002/jev2.12144 PMC8681215 34919343

[9] Mallia A, Gianazza E, Zoanni B, Brioschi M, Barbieri SS, Banfi C (2020). Proteomics of extracellular vesicles: update on their composition, biological roles and potential use as diagnostic tools in atherosclerotic cardiovascular diseases. Diagnostics (Basel).

[10] Brennan K, Martin K, FitzGerald SP, O’Sullivan J, Wu Y, Blanco A, Richardson C, Mc Gee MM (2020). A comparison of methods for the isolation and separation of extracellular vesicles from protein and lipid particles in human serum. Sci Rep.

[11] Tu C, Rudnick PA, Martinez MY, Cheek KL, Stein SE, Slebos RJC, Liebler DC (2010). Depletion of abundant plasma proteins and limitations of plasma proteomics. J Proteome Res.

[12] Cao X, Sandberg A, Araújo JE, Cvetkovski F, Berglund E, Eriksson LE, Pernemalm M (2021). Evaluation of spin columns for human plasma depletion to facilitate MS-based proteomics analysis of plasma. J Proteome Res.

[13] Keshishian H, Burgess MW, Specht H, Wallace L, Clauser KR, Gillette MA, Carr SA (2017). Quantitative, multiplexed workflow for deep analysis of human blood plasma and biomarker discovery by mass spectrometry. Nat Protoc.

[14] Holcar M, Ferdin J, Sitar S, Tušek-Žnidarič M, Dolžan V, Plemenitaš A, Žagar E, Lenassi M (2020). Enrichment of plasma extracellular vesicles for reliable quantification of their size and concentration for biomarker discovery. Sci Rep.

[15] Gámez-Valero A, Monguió-Tortajada M, Carreras-Planella L, Franquesa M, Beyer K, Borràs FE (2016). Size-exclusion chromatography-based isolation minimally alters extracellular vesicles’ characteristics compared to precipitating agents. Sci Rep.

[16] Takov K, Yellon DM, Davidson SM. Comparison of small extracellular vesicles isolated from plasma by ultracentrifugation or size-exclusion chromatography: yield, purity and functional potential. J Extracell Vesicl. 2019; 8:1560809. 10.1080/20013078.2018.1560809 10.1080/20013078.2018.1560809 PMC6327926 30651940

[17] Böing AN, van der Pol E, Grootemaat AE, Coumans FAW, Sturk A, Nieuwland R (2014). Single-step isolation of extracellular vesicles by size-exclusion chromatography. Journal of Extracellular Vesicles.

[18] Vujić T, Schvartz D, Furlani IL, Meister I, González-Ruiz V, Rudaz S, Sanchez J-C (2022). Oxidative stress and extracellular matrix remodeling are signature pathways of extracellular vesicles released upon morphine exposure on human brain microvascular endothelial cells. Cells.

[19] Perez-Riverol Y, Csordas A, Bai J, Bernal-Llinares M, Hewapathirana S, Kundu DJ, Inuganti A, Griss J, Mayer G, Eisenacher M, Pérez E, Uszkoreit J, Pfeuffer J, Sachsenberg T, Yilmaz S, Tiwary S, Cox J, Audain E, Walzer M, Jarnuczak AF, Ternent T, Brazma A, Vizcaíno JA (2019). The PRIDE database and related tools and resources in 2019: improving support for quantification data. Nucleic Acids Res.

[20] George SK, Lauková L, Weiss R, Semak V, Fendl B, Weiss VU, Steinberger S, Allmaier G, Tripisciano C, Weber V (2021). Comparative analysis of platelet-derived extracellular vesicles using flow cytometry and nanoparticle tracking analysis. Int J Mol Sci.

[21] Théry C, Witwer KW, Aikawa E, Alcaraz MJ, Anderson JD, Andriantsitohaina R, Antoniou A, Arab T, Archer F, Atkin-Smith GK, Ayre DC, Bach J-M, Bachurski D, Baharvand H, Balaj L, Baldacchino S, Bauer NN, Baxter AA, Bebawy M, Beckham C, Bedina Zavec A, Benmoussa A, Berardi AC, Bergese P, Bielska E, Blenkiron C, Bobis-Wozowicz S, Boilard E, Boireau W, Bongiovanni A, Borràs FE, Bosch S, Boulanger CM, Breakefield X, Breglio AM, Brennan MÁ, Brigstock DR, Brisson A, Broekman ML, Bromberg JF, Bryl-Górecka P, Buch S, Buck AH, Burger D, Busatto S, Buschmann D, Bussolati B, Buzás EI, Byrd JB, Camussi G, Carter DR, Caruso S, Chamley LW, Chang Y-T, Chen C, Chen S, Cheng L, Chin AR, Clayton A, Clerici SP, Cocks A, Cocucci E, Coffey RJ, Cordeiro-da-Silva A, Couch Y, Coumans FA, Coyle B, Crescitelli R, Criado MF, D’Souza-Schorey C, Das S, Datta Chaudhuri A, de Candia P, De Santana EF, De Wever O, del Portillo HA, Demaret T, Deville S, Devitt A, Dhondt B, Di Vizio D, Dieterich LC, Dolo V, Dominguez Rubio AP, Dominici M, Dourado MR, Driedonks TA, Duarte FV, Duncan HM, Eichenberger RM, Ekström K, EL Andaloussi S, Elie-Caille C, Erdbrügger U, Falcón-Pérez JM, Fatima F, Fish JE, Flores-Bellver M, Försönits A, Frelet-Barrand A, Fricke F, Fuhrmann G, Gabrielsson S, Gámez-Valero A, Gardiner C, Gärtner K, Gaudin R, Gho YS, Giebel B, Gilbert C, Gimona M, Giusti I, Goberdhan DC, Görgens A, Gorski SM, Greening DW, Gross JC, Gualerzi A, Gupta GN, Gustafson D, Handberg A, Haraszti RA, Harrison P, Hegyesi H, Hendrix A, Hill AF, Hochberg FH, Hoffmann KF, Holder B, Holthofer H, Hosseinkhani B, Hu G, Huang Y, Huber V, Hunt S, Ibrahim AG-E, Ikezu T, Inal JM, Isin M, Ivanova A, Jackson HK, Jacobsen S, Jay SM, Jayachandran M, Jenster G, Jiang L, Johnson SM, Jones JC, Jong A, Jovanovic-Talisman T, Jung S, Kalluri R, Kano S, Kaur S, Kawamura Y, Keller ET, Khamari D, Khomyakova E, Khvorova A, Kierulf P, Kim KP, Kislinger T, Klingeborn M, Klinke DJ, Kornek M, Kosanović MM, Kovács ÁF, Krämer-Albers E-M, Krasemann S, Krause M, Kurochkin IV, Kusuma GD, Kuypers S, Laitinen S, Langevin SM, Languino LR, Lannigan J, Lässer C, Laurent LC, Lavieu G, Lázaro-Ibáñez E, Le Lay S, Lee M-S, Lee YXF, Lemos DS, Lenassi M, Leszczynska A, Li IT, Liao K, Libregts SF, Ligeti E, Lim R, Lim SK, Linē A, Linnemannstöns K, Llorente A, Lombard CA, Lorenowicz MJ, Lörincz ÁM, Lötvall J, Lovett J, Lowry MC, Loyer X, Lu Q, Lukomska B, Lunavat TR, Maas SL, Malhi H, Marcilla A, Mariani J, Mariscal J, Martens-Uzunova ES, Martin-Jaular L, Martinez MC, Martins VR, Mathieu M, Mathivanan S, Maugeri M, McGinnis LK, McVey MJ, Meckes DG, Meehan KL, Mertens I, Minciacchi VR, Möller A, Møller Jørgensen M, Morales-Kastresana A, Morhayim J, Mullier F, Muraca M, Musante L, Mussack V, Muth DC, Myburgh KH, Najrana T, Nawaz M, Nazarenko I, Nejsum P, Neri C, Neri T, Nieuwland R, Nimrichter L, Nolan JP, Nolte-’t Hoen EN, Noren Hooten N, O’Driscoll L, O’Grady T, O’Loghlen A, Ochiya T, Olivier M, Ortiz A, Ortiz LA, Osteikoetxea X, Østergaard O, Ostrowski M, Park J, Pegtel DM, Peinado H, Perut F, Pfaffl MW, Phinney DG, Pieters BC, Pink RC, Pisetsky DS, Pogge von Strandmann E, Polakovicova I, Poon IK, Powell BH, Prada I, Pulliam L, Quesenberry P, Radeghieri A, Raffai RL, Raimondo S, Rak J, Ramirez MI, Raposo G, Rayyan MS, Regev-Rudzki N, Ricklefs FL, Robbins PD, Roberts DD, Rodrigues SC, Rohde E, Rome S, Rouschop KM, Rughetti A, Russell AE, Saá P, Sahoo S, Salas-Huenuleo E, Sánchez C, Saugstad JA, Saul MJ, Schiffelers RM, Schneider R, Schøyen TH, Scott A, Shahaj E, Sharma S, Shatnyeva O, Shekari F, Shelke GV, Shetty AK, Shiba K, Siljander PR-M, Silva AM, Skowronek A, Snyder OL, Soares RP, Sódar BW, Soekmadji C, Sotillo J, Stahl PD, Stoorvogel W, Stott SL, Strasser EF, Swift S, Tahara H, Tewari M, Timms K, Tiwari S, Tixeira R, Tkach M, Toh WS, Tomasini R, Torrecilhas AC, Tosar JP, Toxavidis V, Urbanelli L, Vader P, van Balkom BW, van der Grein SG, Van Deun J, van Herwijnen MJ, Van Keuren-Jensen K, van Niel G, van Royen ME, van Wijnen AJ, Vasconcelos MH, Vechetti IJ, Veit TD, Vella LJ, Velot É, Verweij FJ, Vestad B, Viñas JL, Visnovitz T, Vukman KV, Wahlgren J, Watson DC, Wauben MH, Weaver A, Webber JP, Weber V, Wehman AM, Weiss DJ, Welsh JA, Wendt S, Wheelock AM, Wiener Z, Witte L, Wolfram J, Xagorari A, Xander P, Xu J, Yan X, Yáñez-Mó M, Yin H, Yuana Y, Zappulli V, Zarubova J, Žėkas V, Zhang J, Zhao Z, Zheng L, Zheutlin AR, Zickler AM, Zimmermann P, Zivkovic AM, Zocco D, Zuba-Surma EK. Minimal information for studies of extracellular vesicles 2018 (MISEV2018): a position statement of the International Society for Extracellular Vesicles and update of the MISEV2014 guidelines. J Extracell Ves. 2018;7:1535750 10.1080/20013078.2018.1535750 10.1080/20013078.2018.1535750 PMC6322352 30637094

[22] Keerthikumar S, Chisanga D, Ariyaratne D, Saffar HA, Anand S, Zhao K, Samuel M, Pathan M, Jois M, Chilamkurti N, Gangoda L, Mathivanan S (2016). ExoCarta: a web-based compendium of exosomal cargo. J Mol Biol.

[23] Kalra H, Simpson RJ, Ji H, Aikawa E, Altevogt P, Askenase P, Bond VC, Borràs FE, Breakefield X, Budnik V, Buzas E, Camussi G, Clayton A, Cocucci E, Falcon-Perez JM, Gabrielsson S, Gho YS, Gupta D, Harsha HC, Hendrix A, Hill AF, Inal JM, Jenster G, Krämer-Albers E-M, Lim SK, Llorente A, Lötvall J, Marcilla A, Mincheva-Nilsson L, Nazarenko I, Nieuwland R, Hoen ENMN-’t, Pandey A, Patel T, Piper MG, Pluchino S, Prasad TSK, Rajendran L, Raposo G, Record M, Reid GE, Sánchez-Madrid F, Schiffelers RM, Siljander P, Stensballe A, Stoorvogel W, Taylor D, Thery C, Valadi H, Balkom BWM van, Vázquez J, Vidal M, Wauben MHM, Yáñez-Mó M, Zoeller M, Mathivanan S. Vesiclepedia: a compendium for extracellular vesicles with continuous community annotation. PLOS Biolog. 2012;10 e1001450. 10.1371/journal.pbio.1001450 10.1371/journal.pbio.1001450 PMC3525526 23271954

[24] Karimi N, Cvjetkovic A, Jang SC, Crescitelli R, Hosseinpour Feizi MA, Nieuwland R, Lötvall J, Lässer C (2018). Detailed analysis of the plasma extracellular vesicle proteome after separation from lipoproteins. Cell Mol Life Sci.

[25] Vanderboom PM, Dasari S, Ruegsegger GN, Pataky MW, Lucien F, Heppelmann CJ, Lanza IR, Nair KS. A size-exclusion-based approach for purifying extracellular vesicles from human plasma. Cell Rep Meth. 2021;1:100055. 10.1016/j.crmeth.2021.100055 10.1016/j.crmeth.2021.100055 PMC8336930 34355211

[26] Berckmans RJ Extracellular vesicles and coagulation in blood from healthy humans revisited. J Extracel Ves. 10.1080/20013078.2019.1688936 PMC6853244 31762964

[27] Zifkos K, Dubois C, Schäfer K. Extracellular vesicles and thrombosis: update on the clinical and experimental evidence. Int J Mol Sci. 2021. 10.3390/ijms22179317 PMC8431093 34502228

[28] van Niel G, D’Angelo G, Raposo G (2018). Shedding light on the cell biology of extracellular vesicles. Nat Rev Mol Cell Biol.

[29] Diaz Lozano IM, Sork H, Stone VM, Eldh M, Cao X, Pernemalm M, Gabrielsson S, Flodström-Tullberg M. Proteome profiling of whole plasma and plasma-derived extracellular vesicles facilitates the detection of tissue biomarkers in the non-obese diabetic mouse. Front Endocrinol. 202213. 10.3389/fendo.2022.971313 PMC9563222 36246930

[30] de Menezes-Neto A, Sáez MJF, Lozano-Ramos I, Segui-Barber J, Martin-Jaular L, Ullate JME, Fernandez-Becerra C, Borrás FE, del Portillo HA (2015). Size-exclusion chromatography as a stand-alone methodology identifies novel markers in mass spectrometry analyses of plasma-derived vesicles from healthy individuals. J Extracel Ves.

[31] Yuana Y, Böing AN, Grootemaat AE, van der Pol E, Hau CM, Cizmar P, Buhr E, Sturk A, Nieuwland R (2015). Handling and storage of human body fluids for analysis of extracellular vesicles. J Extracel Ves.

[32] Coumans FAW, Brisson AR, Buzas EI, Dignat-George F, Drees EEE, El-Andaloussi S, Emanueli C, Gasecka A, Hendrix A, Hill AF, Lacroix R, Lee Y, van Leeuwen TG, Mackman N, Mäger I, Nolan JP, van der Pol E, Pegtel DM, Sahoo S, Siljander PRM, Sturk G, de Wever O, Nieuwland R (2017). Methodological guidelines to study extracellular vesicles. Circ Res.

[33] Turner NP, Abeysinghe P, Kwan Cheung KA, Vaswani K, Logan J, Sadowski P, Mitchell MD (2022). A comparison of blood plasma small extracellular vesicle enrichment strategies for proteomic analysis. Proteomes.

[34] Kalra H, Adda CG, Liem M, Ang C-S, Mechler A, Simpson RJ, Hulett MD, Mathivanan S (2013). Comparative proteomics evaluation of plasma exosome isolation techniques and assessment of the stability of exosomes in normal human blood plasma. Proteomics.

[35] Karimi N, Dalirfardouei R, Dias T, Lötvall J, Lässer C. Tetraspanins distinguish separate extracellular vesicle subpopulations in human serum and plasma – contributions of platelet extracellular vesicles in plasma samples. J Extracel Ves. 2022;11:e12213. 10.1002/jev2.12213 10.1002/jev2.12213 PMC9077141 35524458

