# Supplementary material for: Depletion of abundant plasma proteins for extracellular vesicle proteome characterization: benefits and pitfalls
Source: Anal Bioanal Chem. 2023 Apr 18;415(16):3177–87. doi: 10.1007/s00216-023-04684-w (PMC10287573; doi:10.1007/s00216-023-04684-w)
Supplement: Supplementary file 1 — Supplementary file1 (PDF 440 KB) [file 216_2023_4684_MOESM1_ESM.pdf]

Depletion of abundant plasma proteins for extracellular vesicle proteome characterization: benefits and pitfalls

Sandrine Reymond[1]*, Lyssia Gruaz[1] and Jean-Charles Sanchez[1*]

[1] Department of Medicine, Faculty of Medicine, University of Geneva, Geneva, Switzerland

*corresponding author e-mail: sandrine.reymond@unige.ch

**Top 20 of most abundant proteins in undepleted plasma**		**Top 20 of most abundant proteins in depleted plasma**	
PG.Genes	PG.ProteinDescriptions	PG.Genes	PG.ProteinDescriptions
**ALB**	**Serum albumin**	**ALB**	**Serum albumin**
**A2M**	**Alpha-2-macroglobulin**	HPX	Hemopexin
HPX	Hemopexin	SERPINA3	Alpha-1-antichymotrypsin
**APOA1**	**Apolipoprotein A-I**	CP	Ceruloplasmin
**TF**	**Serotransferrin**	AMBP	Protein AMBP
**FGB**	**Fibrinogen beta chain**	**APOA1**	**Apolipoprotein A-I**
**IGHA1**	**Immunoglobulin heavy constant alpha 1**	C3	Complement C3
**SERPINA1**	**Alpha-1-antitrypsin**	GC	Vitamin D-binding protein
**IGHG1**	**Immunoglobulin heavy constant gamma 1**	PLG	Plasminogen
**HP**	**Haptoglobin**	VTN	Vitronectin
**IGHM**	**Immunoglobulin heavy constant mu**	AHSG	Alpha-2-HS-glycoprotein
**IGKC**	**Immunoglobulin kappa constant**	APOA4	Apolipoprotein A-IV
CP	Ceruloplasmin	ITIH2	Inter-alpha-trypsin inhibitor heavy chain H2
**FGG**	**Fibrinogen gamma chain**	A1BG	Alpha-1B-glycoprotein
**IGHG2**	**Immunoglobulin heavy constant gamma 2**	CFH	Complement factor H
AMBP	Protein AMBP	C1QC	Complement C1q subcomponent subunit C
SERPINA3	Alpha-1-antichymotrypsin	APOB	Apolipoprotein B-100
VTN	Vitronectin	FN1	Fibronectin
**FGA**	**Fibrinogen alpha chain**	SERPING1	Plasma protease C1 inhibitor
GC	Vitamin D-binding protein	SERPINC1	Antithrombin-III

Figure S1 Top 20 of most abundant proteins in depleted plasma using High Select^{TM} Top14 Abundant Protein Depletion Midi Spin Columns (ThermoScientific, Waltham, MA, USA) and on non-depleted plasma by LC-MS/MS. In bold are proteins targeted for depletion: serum albumin, IgG, IgA, IgM, IgD, IgE, kappa and lambda light chains, alpha-1-acidglycoprotein, alpha-1-antitrypsin, alpha-2-macroglobulin, apolipoprotein A-I, fibrinogen, haptoglobin, and serotransferrin

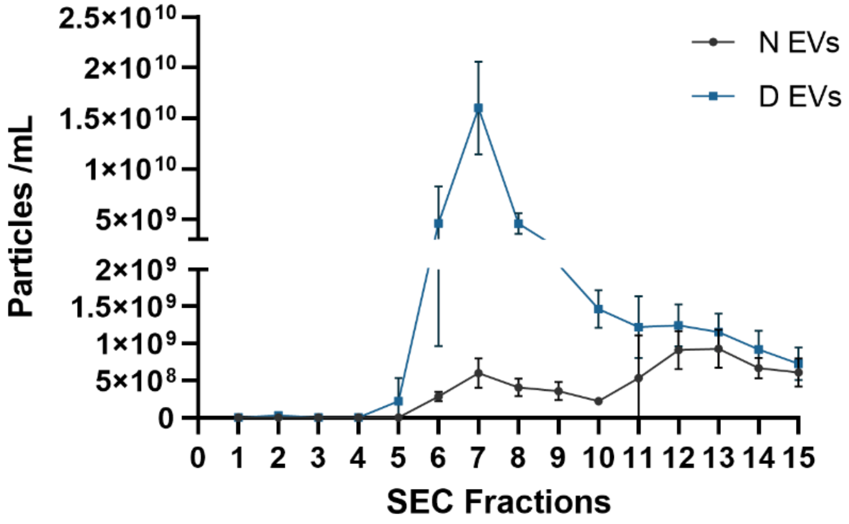

Figure S2 Concentrations of particles in the SEC fractions were determined with Nanoparticle Tracking Analysis in scatter mode (s-NTA) (n=3) and the results are presented as the mean ± SD

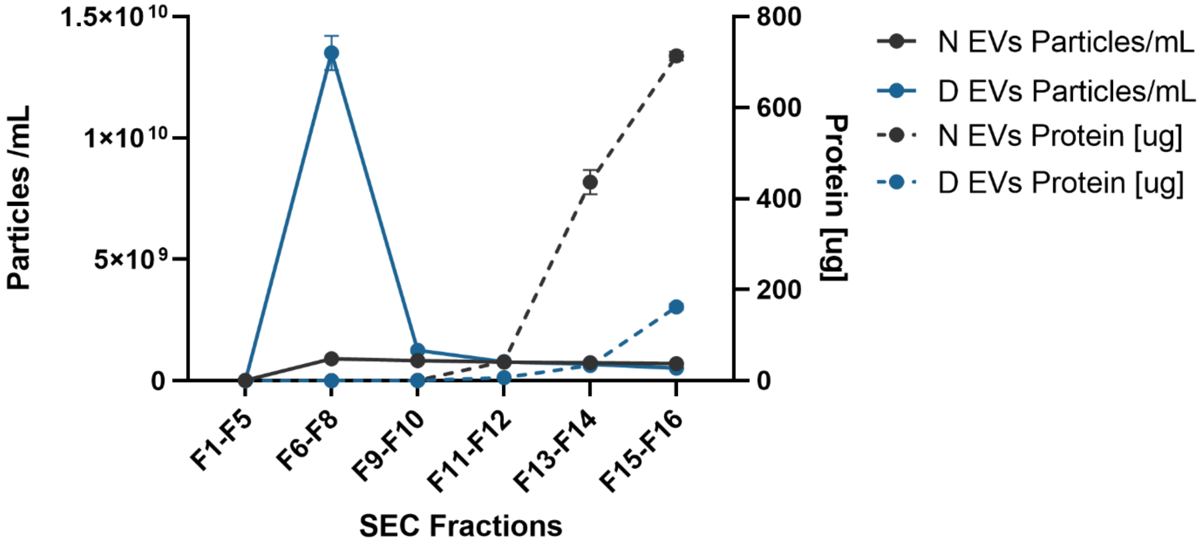

Figure S3 EVs isolated from non-depleted plasma (N EVs) are represented in black and depleted plasma (DC EVs) in dark blue. Size-exclusion chromatography (SEC) fraction profiles of NTA-measured particles concentrations (solid line) and total protein quantity measured using Bradford assay (dashed line) to illustrate separation of EVs from plasma proteins. F1 to F16 correspond to 200-µL SEC fractions, which were pooled for analysis

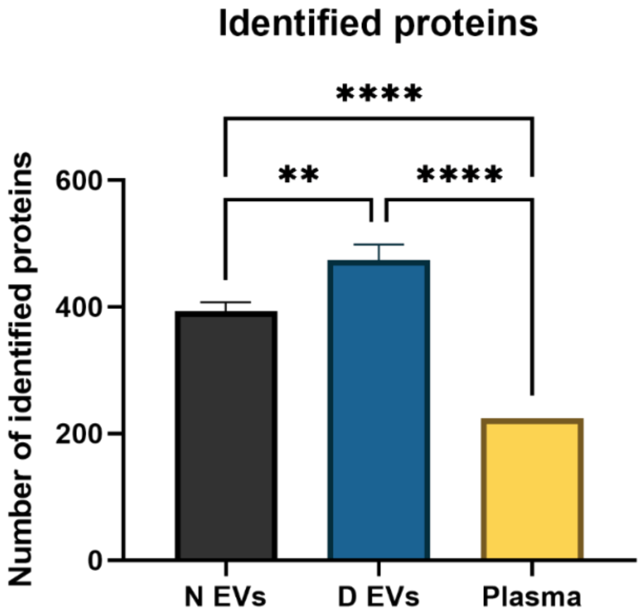

Figure S4 Number of identified proteins by mass spectrometry for extracellular vesicles isolated from non-depleted plasma (N EVs) and depleted plasma (D EVs) as well as crude plasma. One-way ANOVA, Turkey test, ** p-value <0.005, **** p-value < 0.0001

Top 20 most abundant proteins in N EVs			Top 20 most abundant proteins in D EVs		
Gene name	Protein name	Mean	Gene name	Protein name	Mean
**IGHM**	Immunoglobulin heavy constant mu	2.91E+07	**ALB**	Serum albumin	7.44E+06
VWF	von Willebrand factor	2.50E+07	APOB	Apolipoprotein B-100	4.41E+06
**ALB**	Serum albumin	9.34E+06	VWF	von Willebrand factor	3.80E+06
**IGKC**	Immunoglobulin kappa constant	5.53E+06	APOE	Apolipoprotein E	2.79E+06
FCN3	Ficolin-3	4.94E+06	AHSG	Alpha-2-HS-glycoprotein	2.66E+06
**IGLL5**	Immunoglobulin lambda-like polypeptide 5	4.30E+06	**IGHM**	Immunoglobulin heavy constant mu	2.34E+06
**A2M**	Alpha-2-macroglobulin	3.58E+06	AMBP	Protein AMBP	2.32E+06
SPTA1	Spectrin alpha chain, erythrocytic 1	3.45E+06	KNG1	Kininogen-1	1.77E+06
APOB	Apolipoprotein B-100	3.04E+06	C3	Complement C3	1.69E+06
CD5L	CD5 antigen-like	2.18E+06	CLU	Clusterin	1.46E+06
APOE	Apolipoprotein E	2.07E+06	ITIH2	Inter-alpha-trypsin inhibitor heavy chain H2	1.25E+06
LPA	Apolipoprotein(a)	1.95E+06	HRG	Histidine-rich glycoprotein	1.11E+06
HBB	Hemoglobin subunit beta	1.68E+06	LPA	Apolipoprotein(a)	1.05E+06
JCHAIN	Immunoglobulin J chain	1.65E+06	VTN	Vitronectin	8.51E+05
**IGHA1**	Immunoglobulin heavy constant alpha 1	1.59E+06	**IGKC**	Immunoglobulin kappa constant	7.81E+05
ITGB3	Integrin beta-3	1.46E+06	GSN	Gelsolin	7.50E+05
SLC2A1	Solute carrier family 2, facilitated glucose transporter member 1	1.20E+06	ITIH4	Inter-alpha-trypsin inhibitor heavy chain H4	7.15E+05
**IGHV3-72**	Immunoglobulin heavy variable 3-72	1.14E+06	C9	Complement component C9	7.01E+05
**IGKV3-20**	Immunoglobulin kappa variable 3-20	1.09E+06	DEFA1	Neutrophil defensin 1	6.72E+05
**IGKV3D-11**	Immunoglobulin kappa variable 3D-11	1.05E+06	SERPINA3	Alpha-1-antichymotrypsin	6.66E+05

Figure S5 Top 20 of most abundant proteins in undepleted plasma-derived EVs (N EVs) and depleted plasma-derived EVs (D EVs) by LC-MS/MS. Keratins were removed as from exogenous source

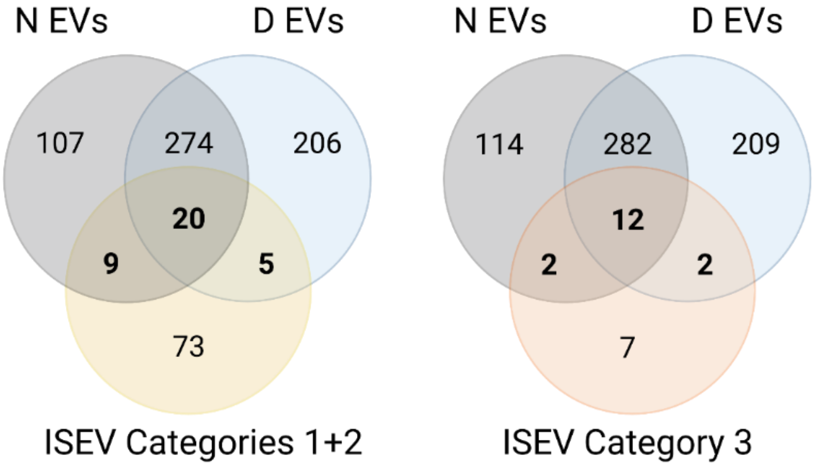

Figure S6 Venn diagram of identified proteins for N and D EVs and proteins from categories 1, 2 and 3 from Table 3 of the Minimal Information for Studies of EVs (MISEV2018) for protein content-based EV characterization[1]. Categories 1 and 2 are used as EV-associated proteins, while the third category contains non-EV co-isolated components. Comparison was performed through the adaptation of a detailed proteins list provided by the study of Muraoka et al[2].

[1] Théry C, Witwer KW, Aikawa E, Alcaraz MJ, Anderson JD, Andriantsitohaina R, Antoniou A, Arab T, Archer F, Atkin-Smith GK, Ayre DC, Bach J-M, Bachurski D, Baharvand H, Balaj L, Baldacchino S, Bauer NN, Baxter AA, Bebawy M, Beckham C, Bedina Zavec A, Benmoussa A, Berardi AC, Bergese P, Bielska E, Blenkiron C, Bobis-Wozowicz S, Boilard E, Boireau W, Bongiovanni A, Borràs FE, Bosch S, Boulanger CM, Breakefield X, Breglio AM, Brennan MÁ, Brigstock DR, Brisson A, Broekman ML, Bromberg JF, Bryl-Górecka P, Buch S, Buck AH, Burger D, Busatto S, Buschmann D, Bussolati B, Buzás EI, Byrd JB, Camussi G, Carter DR, Caruso S, Chamley LW, Chang Y-T, Chen C, Chen S, Cheng L, Chin AR, Clayton A, Clerici SP, Cocks A, Cocucci E, Coffey RJ, Cordeiro-da-Silva A, Couch Y, Coumans FA, Coyle B, Crescitelli R, Criado MF, D'Souza-Schorey C, Das S, Datta Chaudhuri A, de Candia P, De Santana EF, De Wever O, del Portillo HA, Demaret T, Deville S, Devitt A, Dhondt B, Di Vizio D, Dieterich LC, Dolo V, Dominguez Rubio AP, Dominici M, Dourado MR, Driedonks TA, Duarte FV, Duncan HM, Eichenberger RM, Ekström K, EL Andaloussi S, Elie-Caille C, Erdbrügger U, Falcón-Pérez JM, Fatima F, Fish JE, Flores-Bellver M, Försönits A, Frelet-Barrand A, Fricke F, Fuhrmann G, Gabrielsson S, Gámez-Valero A, Gardiner C, Gärtner K, Gaudin R, Gho YS, Giebel B, Gilbert C, Gimona M, Giusti I, Goberdhan DC, Görgens A, Gorski SM, Greening DW, Gross JC, Gualerzi A, Gupta GN, Gustafson D, Handberg A, Haraszti RA, Harrison P, Hegyesi H, Hendrix A, Hill AF, Hochberg FH, Hoffmann KF, Holder B, Holthofer H, Hosseinkhani B, Hu G, Huang Y, Huber V, Hunt S, Ibrahim AG-E, Ikezu T, Inal JM, Isin M, Ivanova A, Jackson HK, Jacobsen S, Jay SM, Jayachandran M, Jenster G, Jiang L, Johnson SM, Jones JC, Jong A, Jovanovic-Talisman T, Jung S, Kalluri R, Kano S, Kaur S, Kawamura Y, Keller ET, Khamari D, Khomyakova E, Khvorova A, Kierulf P, Kim KP, Kislinger T, Klingeborn M, Klinke DJ, Kornek M, Kosanović MM, Kovács ÁF, Krämer-Albers E-M, Krasemann S, Krause M, Kurochkin IV, Kusuma GD, Kuypers S, Laitinen S, Langevin SM, Languino LR, Lannigan J, Lässer C, Laurent LC, Lavieu G, Lázaro-Ibáñez E, Le Lay S, Lee M-S, Lee YXF, Lemos DS, Lenassi M, Leszczynska A, Li IT, Liao K, Libregts SF, Ligeti E, Lim R, Lim SK, Linē A, Linnemannstöns K, Llorente A, Lombard CA, Lorenowicz MJ, Lörincz ÁM, Lötvall J, Lovett J, Lowry MC, Loyer X, Lu Q, Lukomska B, Lunavat TR, Maas SL, Malhi H, Marcilla A, Mariani J, Mariscal J, Martens-Uzunova ES, Martin-Jaular L, Martinez MC, Martins VR, Mathieu M, Mathivanan S, Maugeri M, McGinnis LK, McVey MJ, Meckes DG, Meehan KL, Mertens I, Minciacchi VR, Möller A, Møller Jørgensen M, Morales-Kastresana A, Morhayim J, Mullier F, Muraca M, Musante L, Mussack V, Muth DC, Myburgh KH, Najrana T, Nawaz M, Nazarenko I, Nejsum P, Neri C, Neri T, Nieuwland R, Nimrichter L, Nolan JP, Nolte-'t Hoen EN, Noren Hooten N, O'Driscoll L, O'Grady T, O'Loghlen A, Ochiya T, Olivier M, Ortiz A, Ortiz LA, Osteikoetxea X, Østergaard O, Ostrowski M, Park J, Pegtel DM, Peinado H, Perut F, Pfaffl MW, Phinney DG, Pieters BC, Pink RC, Pisetsky DS, Pogge von Strandmann E, Polakovicova I, Poon IK, Powell BH, Prada I, Pulliam L, Quesenberry P, Radeghieri A, Raffai RL, Raimondo S, Rak J, Ramirez MI, Raposo G, Rayyan MS, Regev-Rudzki N, Ricklefs FL, Robbins PD, Roberts DD, Rodrigues SC, Rohde E, Rome S, Rouschop KM, Rughetti A, Russell AE, Saá P, Sahoo S, Salas-Huenuleo E, Sánchez C, Saugstad JA, Saul MJ, Schiffelers RM, Schneider R, Schøyen TH, Scott A, Shahaj E, Sharma S, Shatnyeva O, Shekari F, Shelke GV, Shetty AK, Shiba K, Siljander PR-M, Silva AM, Skowronek A, Snyder OL, Soares RP, Sódar BW, Soekmadji C, Sotillo J, Stahl PD, Stoorvogel W, Stott SL, Strasser EF, Swift S, Tahara H, Tewari M, Timms K, Tiwari S, Tixeira R, Tkach M, Toh WS, Tomasini R, Torrecilhas AC, Tosar JP, Toxavidis V, Urbanelli L, Vader P, van Balkom BW, van der Grein SG, Van Deun J, van Herwijnen MJ, Van Keuren-Jensen K, van Niel G, van Royen ME, van Wijnen AJ, Vasconcelos MH, Vechetti IJ, Veit TD, Vella LJ, Velot É, Verweij FJ, Vestad B, Viñas JL, Visnovitz T, Vukman KV, Wahlgren J, Watson DC, Wauben MH, Weaver A, Webber JP, Weber V, Wehman AM, Weiss DJ, Welsh JA, Wendt S, Wheelock AM, Wiener Z, Witte L, Wolfram J, Xagorari A, Xander P, Xu J, Yan X, Yáñez-Mó M, Yin H, Yuana Y, Zappulli V, Zarubova J, Žėkas V, Zhang J, Zhao Z, Zheng L, Zheutlin AR, Zickler AM, Zimmermann P, Zivkovic AM, Zocco D, Zuba-Surma EK (2018) Minimal information for studies of extracellular vesicles 2018 (MISEV2018): a position statement of the International Society for Extracellular Vesicles and update of the MISEV2014 guidelines. Journal of Extracellular Vesicles 7:1535750. https://doi.org/10.1080/20013078.2018.1535750

[2] Muraoka S, Jedrychowski MP, Tatebe H, DeLeo AM, Ikezu S, Tokuda T, Gygi SP, Stern RA, Ikezu T (2019) Proteomic Profiling of Extracellular Vesicles Isolated From Cerebrospinal Fluid of Former National Football League Players at Risk for Chronic Traumatic Encephalopathy. Frontiers in Neuroscience 13
